# AutoFlow® versus volume-controlled ventilation for laparoscopic gynecological surgery using LMA® ProSeal™: a randomized controlled trial

**DOI:** 10.1186/s12871-021-01406-6

**Published:** 2021-06-28

**Authors:** Toshiyuki Nakanishi, Seishi Sakamoto, Manabu Yoshimura, Takashi Toriumi

**Affiliations:** 1 Department of Anesthesiology, Japan Community Healthcare Organization Tokuyama Central Hospital, Shunan, Japan; 2 Present address: Department of Anesthesiology and Intensive Care Medicine, Nagoya City University Graduate School of Medical Sciences, Kawasumi 1, Mizuho-cho, Mizuho-ku, Nagoya, Japan

**Keywords:** Airway management, Laryngeal masks, Mechanical ventilation

## Abstract

**Background:**

During laparoscopic gynecological surgery, increased peak airway pressure (PAWP) can cause airway leak upon ventilation with the LMA® ProSeal™. We hypothesized that compared with the use of volume-controlled ventilation (VCV), the use of the AutoFlow® mode would decrease PAWP and airway leak during laparoscopic gynecological surgery with LMA ProSeal.

**Methods:**

This single-center, randomized, controlled trial allocated 80 adult women undergoing elective laparoscopic gynecological surgery to one of two groups, namely, the AutoFlow group or the VCV group. Ventilation settings for both groups were 8 ml/kg of tidal volume and 5 cmH_2_O of positive end-expiratory pressure, and respiratory rate was adjusted to maintain end-tidal carbon dioxide at 35–40 mmHg. Airway leak, PAWP, and other ventilatory parameters and vital signs were recorded at four timepoints (1, 1 min after insertion of the gastric tube; 2, 2 min after intravenous administration of rocuronium 0.6–0.8 mg/kg; 3, 1 min after initiation of pneumoperitoneum; and 4, 1 min after changing to the Trendelenburg position). The primary outcome was PAWP during pneumoperitoneum and in the Trendelenburg position, whereas the secondary outcomes included PAWP at other timepoints and airway leak development. We used the Mann–Whitney U test for PAWP and Fisher’s exact test for comparing airway leak among the groups.

**Results:**

Data from 40 patients in the AutoFlow group and 39 in the VCV group were used for analysis. PAWP at pneumoperitoneum pressure and in the Trendelenburg position was significantly lower in the AutoFlow group than in the VCV group [median (interquartile range), 16 (15–18) cmH_2_O vs. 18 (17–19) cmH_2_O; *P* < 0.001]. Similarly, patients in the AutoFlow group showed lower PAWP at the other three timepoints measured. Airway leak occurred in four patients in the AutoFlow group and in two patients in the VCV group; however, this incidence was not significantly different (*P* = 0.68).

**Conclusions:**

Even though AutoFlow ventilation decreased PAWP, it did not reduce the incidence of airway leak compared with VCV during laparoscopic gynecological surgery with the LMA ProSeal.

**Trial registration:**

UMIN Clinical Trials Registry, identifier UMIN000023173.

**Supplementary Information:**

The online version contains supplementary material available at 10.1186/s12871-021-01406-6.

## Introduction

LMA® ProSeal™ (pLMA) is the oldest second-generation supraglottic airway device with a gastric tube channel. In laparoscopic gynecological surgery, endotracheal tubes have been used most commonly, but the use of pLMA has been reported as a useful alternative [1–3]. During such procedures, the need for pneumoperitoneum and the placement of the patient in the Trendelenburg position raise patients’ diaphragms, which results in decreased lung compliance and elevated peak airway pressure (PAWP). Furthermore, during positive pressure ventilation using supraglottic airway devices, elevated PAWP can lead to airway leaks and to subsequent sequelae such as hypoventilation, gastric insufflation, and operating room pollution [4]. Thus, avoiding PAWP elevation and airway leak is essential while using supraglottic airway devices during laparoscopic gynecological surgery.

AutoFlow® is one of the dual-controlled ventilation (DCV) modes, which uses a decelerative flow pattern like pressure-controlled ventilation (PCV) to adjust inspiratory pressure and to deliver a defined tidal volume at every breath. Thus, DCV has the advantages of both PCV and volume-controlled ventilation (VCV) and can, therefore, prevent PAWP elevation and ensure target tidal volume even when lung compliance changes, such as during laparoscopy and in the Trendelenburg position [5]. During laparoscopic surgery in the Trendelenburg position with tracheal intubation, DCV has been reported to result in lower PAW*P* values than VCV [5, 6]. Thus, suppressing PAWP with AutoFlow ventilation may prevent airway leak during laparoscopic gynecological surgery with pLMA. However, whether DCV can effectively regulate PAWP, avoid airway leak, and maintain adequate tidal volume during laparoscopic surgery with supraglottic airway devices is unknown. Therefore, we hypothesized that, compared with VCV, AutoFlow would decrease PAWP and airway leak during laparoscopic gynecological surgery with pLMA.

## Methods

This prospective, randomized, parallel-group, single-blinded clinical trial was approved by the Tokuyama Central Hospital Institutional Review Board (K243–20160511), was registered in the UMIN Clinical Trials Registry (identifier: UMIN000023173, date 14/07/2016), and was conducted at the Japan Community Healthcare Organization Tokuyama Central Hospital, a tertiary hospital in Japan, between July 2016 and August 2017. Written informed consent for study participation was obtained from all the participants. All methods were performed in accordance with the CONSORT 2010 statement.

### Study participants

We screened and recruited 80 adult women undergoing elective laparoscopic gynecological surgery (adnexectomy, cystectomy, hysterectomy, myomectomy, ovarian drilling, and salpingectomy), aged 20–80 years, and with American Society of Anesthesiologists physical status (ASA-PS) 1–2. Patients who were thought to be unsuitable for management with pLMA, such as those with morbid obesity (body mass index > 35 kg/m^2^), who have not fasted, or with gastroesophageal reflux disease, were excluded from the study.

Patients were randomly allocated by the principal investigator (TN) to one of two groups, i.e., the AutoFlow group or the VCV group, in a 1:1 ratio with a block size of 2 and by using a computer-generated randomization method. During the procedure, all patients were monitored using an electrocardiogram, non-invasive blood pressure measurement, and pulse oximetry in the operating room. In patients with no relevant contraindications, epidural catheters were inserted for intraoperative and postoperative analgesia. After 3 min of pre-oxygenation, anesthesia was induced with intravenous administration of 2 μg/kg of fentanyl and 2 mg/kg of propofol. This was done without using a neuromuscular blocking drug, as per our hospital’s standard method. Continuous intravenous infusion of remifentanil at 0.25 μg/kg/min was used in patients who could not be provided epidural analgesia. After the loss of consciousness, an attending anesthesiologist performed mask ventilation and maintained requisite oxygenation. Next, the attending anesthesiologist inserted the pLMA and inflated the cuff with air (12 ml for size 3 and 15 ml for size 4). The size of the pLMA used was selected according to the manufacturer’s manual, which recommends size 3 for body weight < 50 kg and size 4 for weight ≥ 50 kg. In patients who were not provided epidural anesthesia, an ultrasound-guided peripheral nerve block (rectus sheath block and posterior transversus abdominis plane block) was preoperatively administered using 40–50 ml of 0.3% ropivacaine.

We assessed pLMA insertion by performing a bubble test and inserting a gastric tube and by measuring oropharyngeal leak pressure (OLP) and fiberoptic score (FOS), as described below [7]. In the bubble test, we injected a water-soluble jelly into the gastric tube channel of the pLMA and initiated positive pressure ventilation. If bubbles were visible, we defined the bubble test to be positive, implying that the pLMA was inserted in an improper position. Next, we inserted a 14 Fr gastric tube and estimated the ease of gastric tube insertion. After inserting the gastric tube, we raised fresh gas flow to 3 l/min with the adjustable pressure-limiting valve set at 40 cmH_2_O and monitored equilibrated pressure as OLP [8]. Finally, using a flexible bronchoscope, we observed the patient’s larynx through the ventilation port of the pLMA and graded FOS as described previously (4, only vocal cords were seen; 3, vocal cords and posterior epiglottis were seen; 2, vocal cords and anterior epiglottis were seen; 1, vocal cords were not seen but function adequate; and 0, function failure) [9]. The position of the pLMA was modified if the bubble test was positive, gastric tube insertion was difficult, OLP < 20 cmH_2_O, or if FOS grade 0 or 1 was observed. Finally, if the attending anesthesiologist assessed pLMA as inappropriate even after repositioning, the patient was discontinued from further participation. If the overall fitting test was good, 0.6–0.8 mg/kg of rocuronium was administered intravenously before surgical incision.

The anesthesia equipment used in all patients was the Apollo® (Dräger, Lübeck, Germany). AutoFlow ventilation mode was used in the AutoFlow group, whereas the VCV mode was used in the VCV group during the overall surgical procedure. Tidal volume was set at 8 ml/kg of ideal body weight, and a positive end-expiratory pressure of 5 cmH_2_O was used in all patients. Inspiratory–expiratory time ratio was set at 1:2, and respiratory rate was adjusted to 12–16 per minute to maintain end-tidal carbon dioxide (EtCO_2_) at 35–40 mmHg. Fractional inspired oxygen (FiO_2_) was set at 0.3–0.45 with 3 l/min of fresh gas flow to maintain SpO_2_ ≥ 94%. Pneumoperitoneum pressure was set at 10 mmHg of intra-abdominal pressure using CO_2_, and the Trendelenburg position was achieved by tilting the patient 15°–18° head down. General anesthesia was maintained in all patients with sevoflurane and intravenous fentanyl and rocuronium. For intraoperative analgesia, patients with epidural catheters received intermittent 3–5 ml epidural infusions of 0.3% ropivacaine, whereas those without epidural catheters were administered a continuous infusion of intravenous remifentanil at 0.1–0.25 μg/kg/min.

Airway leaks in the pLMA, ventilatory parameters, and vital signs were recorded at four timepoints (1, 1 min after insertion of the gastric tube; 2, 2 min after intravenous administration of rocuronium 0.6–0.8 mg/kg; 3, 1 min after initiation of pneumoperitoneum; and 4, 1 min after changing to the Trendelenburg position). The primary outcome was PAWP at pneumoperitoneum and in the Trendelenburg position, whereas the secondary outcomes included PAWP at the other timepoints and the development of an airway leak. We defined an air leak as being present if it could be heard obviously or with the use of a stethoscope, and leaks were graded into three levels based on this as obviously audible, audible by neck auscultation with a stethoscope, and inaudible [10]. We also evaluated whether target tidal volume could be maintained and compared the same between the two groups by calculating the leak fraction, which was defined as the difference between the defined tidal volume and expiratory tidal volume divided by the defined tidal volume.

### Statistical analysis

Based on a pilot study that showed that the difference in PAWP between AutoFlow ventilation and VCV, at both pneumoperitoneum and in the Trendelenburg position, was 2.6 cmH_2_O with a standard deviation of 4, we calculated that 38 patients were needed in each group to detect a significant difference at an alpha error of 0.05 and a beta error of 0.2. To account for the potential dropout of a few cases, we recruited 80 participants for the study, i.e., 40 per group. We used the Mann–Whitney U test to compare continuous variables (PAWP, OLP, FiO_2_, EtCO_2_, end-tidal sevoflurane, respiratory rate, leak fraction, SpO_2_, heart rate, and non-invasive blood pressure); Fisher’s exact test for airway leak, bubble test, and gastric tube insertion; and the Chi-squared test for FOS. We used R, ver. 3.6.3 (R Foundation for Statistical Computing, Vienna, Austria), for all statistical analysis. Data are presented as median [interquartile range]. *P* values of < 0.05 were considered statistically significant.

## Results

**Fig. 1:**
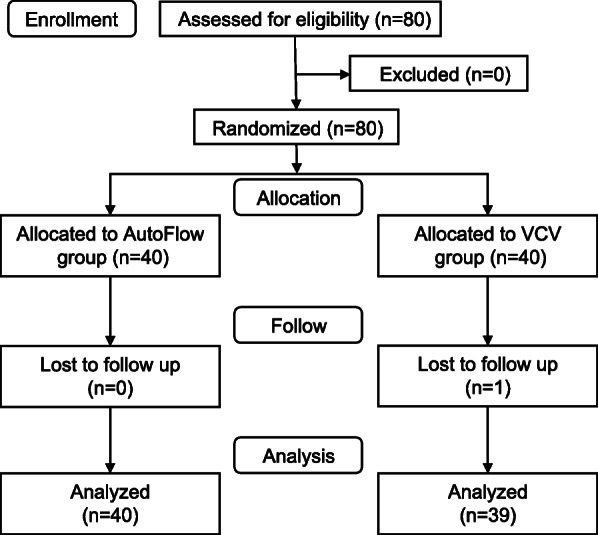
CONSORT diagram of the study participants. VCV: volume-controlled ventilation

All patients could be successfully managed with pLMA throughout the procedure. Among the 40 patients in each group, data for one patient in the VCV group was missing; consequently, the final analysis included only data from 39 patients in the VCV group, along with those from 40 patients in the AutoFlow group (Fig. 1). There were no differences between the two groups in patient characteristics (Table 1) or in the results of the pLMA insertion tests (Table 2).

**Table 1:** Patients’ characteristics and operative data

	AutoFlow group (*n* = 40)	VCV group (*n* = 39)
Age (y)	41 ± 11	45 ± 10
Height (cm)	157 ± 6	159 ± 5
Weight (kg)	54 ± 9	54 ± 8
ASA-PS 1/2	31/9	29/10
Mallampati 1/2/3	15/21/4	21/14/4
pLMA size 3/4	15/25	13/26
Operation duration (min)	118 [66–176]	134 [75–173]
Anesthesia duration (min)	141 [97–215]	171 [100–217]
Type of surgery		
Adnexectomy	10	9
Cystectomy	11	10
LAVH	2	4
Myomectomy	7	4
TLH	8	12
Others^a^	2	0

Data are shown as mean ± SD, number, or median [interquartile range]. ^a^Others include ovarian drilling and salpingectomy.

*VCV* Volume-controlled ventilation, *ASA-PS* American Society of Anesthesiologists physical status, *pLMA* LMA ProSeal, *LAVH* Laparoscopic-assisted vaginal hysterectomy, *TLH* Total laparoscopic hysterectomy

**Table 2:** Fitting tests between the AutoFlow group and the volume-controlled ventilation group

	AutoFlow group (*n* = 40)	VCV group (*n* = 39)	*P* value
Positive bubble test	1 (3)	1 (3)	1
Difficult gastric tube insertion	0 (0)	0 (0)	
OLP (cmH_2_O)	28 [25–34]	27 [25–34]	0.43
FOS 4/3/2	23/6/11	23/10/6	0.29

Data are shown as number (proportion), median [interquartile range], or number.

*VCV* Volume-controlled ventilation, *OLP* Oropharyngeal leak pressure, *FOS* Fiberoptic score

**Fig. 2:**
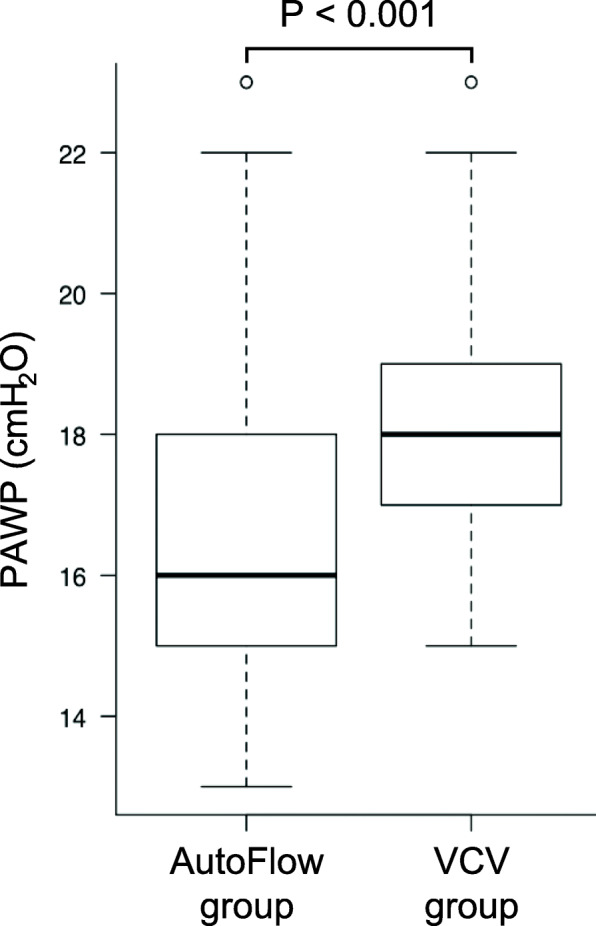
Peak airway pressure at pneumoperitoneum and in the Trendelenburg position. PAWP: peak airway pressure, VCV: volume-controlled ventilation

PAWP at pneumoperitoneum and in the Trendelenburg position was significantly lower in the AutoFlow group compared with that in the VCV group (16 [15–18] cmH_2_O vs. 18 [17–19] cmH_2_O; *P* < 0.001) (Fig. 2). Similarly, patients in the AutoFlow group showed lower PAWP at the other three timepoints measured (Table 3). Airway leak was found in four patients in the AutoFlow group and in two patients in the VCV group; however, this difference was not significant (*P* = 0.68). Leak fraction at pneumoperitoneum and in the Trendelenburg position was comparable between the AutoFlow (− 1.6 [− 5.2 to 0.8] %) and the VCV (− 1.9 [− 4.3 to − 0.6] %, *P* = 0.47) groups. All other ventilatory parameters and vital signs were not different between the groups (Supplementary Table).

**Table 3:** Airway leak of LMA ProSeal and peak airway pressure at four time points

	AutoFlow group (*n* = 40)	VCV group (*n* = 39)	*P* value
1. Airway leak	0 (0)	0 (0)	
2. Airway leak	0 (0)	0 (0)	
3. Airway leak	2 (5)	0 (0)	0.49
4. Airway leak	4 (10)	2 (5)	0.68
1. PAWP (cmH_2_O)	12 [12–13]	13 [12–14]	0.013
2. PAWP (cmH_2_O)	12 [12–13]	13 [13–14]	0.004
3. PAWP (cmH_2_O)	13 [12–15]	16 [12–17]	0.001
4. PAWP (cmH_2_O)	16 [15–18]	18 [17–19]	< 0.001

1, One min after insertion of the gastric tube; 2, Two min after intravenous administration of neuromuscular blocking drug; 3, One min after initiation of pneumoperitoneum; 4, One min after change to the Trendelenburg position. Data are shown as number (proportion), median [interquartile range].

*VCV* Volume-controlled ventilation, *PAWP* Peak airway pressure

**Fig. 3:**
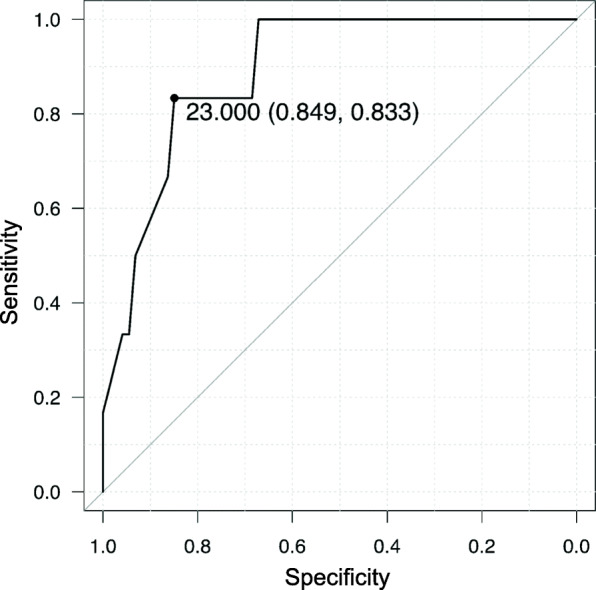
The receiver-operating curve of oropharyngeal leak pressure for the effects on airway leak at pneumoperitoneum and in the Trendelenburg position

To investigate the cause of airway leak occurrence, we compared pLMA insertion results between patients with and without airway leak and found that six patients with airway leak showed lower OLP values than the remaining 73 patients without airway leak (22 [19–23] cmH_2_O vs. 28 [25–34] cmH_2_O; *P* = 0.002). In our study, OLP above PAWP at both pneumoperitoneum and Trendelenburg position was lower for airway leak patients (2 [− 1 to 5] cmH_2_O) compared with that for patients without airway leak (11 [8–17] cmH_2_O) (P = 0.002). The area under the receiver-operating curve of OLP for the effects on airway leak was 0.892 (95% CI 0.787–0.996), and the sum of its sensitivity and specificity was highest (0.833 and 0.849, respectively) at 23 cmH_2_O of OLP (Fig. 3). Furthermore, although FOS was not inferior in those who developed an airway leak compared with those who did not, these six patients with airway leak performed less well in the bubble test (Table 4).

**Table 4:** Fitting tests between patients with or without airway leak

	Leak positive (*n* = 6)	Leak negative (*n* = 73)	*P* value
Positive bubble test	2 (33)	0 (0)	0.005
OLP (cmH_2_O)	22 [19–23]	28 [25–34]	0.002
FOS 4/3/2	2/2/2	44/14/15	0.30

Data are shown as number (proportion), median [interquartile range], or number.

*OLP* Oropharyngeal leak pressure, *FOS* Fiberoptic score

## Discussion

We show that, during laparoscopic gynecological surgery with pLMA, PAWP at pneumoperitoneum and in the Trendelenburg position was significantly lower with AutoFlow ventilation than with VCV. Even though an audible airway leak developed in four patients in the AutoFlow group and in two patients in the VCV group, a similar leak fraction between the groups indicates that target tidal volume was maintained in both groups. Additionally, we found that AutoFlow ventilation achieved lower PAWP than did VCV while maintaining a similar target tidal volume.

Recently, the use of the DCV mode, such as the AutoFlow or PCV-VG (pressure-controlled ventilation volume guaranteed), has been reported to be useful in maintaining lower PAWP during laparoscopic surgeries requiring the Trendelenburg position, compared with VCV, where the patient is ventilated using tracheal intubation [5, 6]. The advantage of DCV over PCV is that the former can maintain adequate tidal volume throughout the procedure, even if lung compliance is altered. More importantly, such altered lung compliance due to pneumoperitoneum or the Trendelenburg position during PCV can result in hypoventilation or hyperventilation, which may increase pulmonary complications compared with VCV [11]. Thus, DCV may be more useful than PCV during laparoscopic gynecological surgery where lung compliance can dynamically change during the procedure; however, the superiority of DCV over PCV in reducing pulmonary complications has not been evaluated. In our study, AutoFlow effectively reduced PAWP and maintained target tidal volume during laparoscopic gynecological surgery with pLMA, which is similar to that reported with tracheal intubation.

Although PAWP was lower in the AutoFlow group, airway leak incidence was higher in this group, though this increase was not different between the two groups. The optimal ventilation mode for supraglottic airway devices has not been established, and several studies have compared the effects of PCV and VCV on ventilatory parameters during positive pressure ventilation with supraglottic airway devices. In a prospective crossover study that compared PCV and VCV in patients ventilated using LMA, PAWP was lower with PCV than with VCV, and the authors suggested that the use of PCV can avoid airway leak, especially in situations with increased respiratory resistance, such as during laparoscopy or surgery in the Trendelenburg position [4]. In a prospective randomized study of laparoscopic gynecological surgery using LMA Classic, at 15 min after pneumoperitoneum, the PAWP values recorded (27.4 ± 4.7 cmH_2_O) were higher than those for OLP (27.0 ± 5.0 cmH_2_O) in patients ventilated with VCV, but not in those with PCV (PAWP, 24.3 ± 3.0 cmH_2_O; OLP, 27.1 ± 4.2 cmH_2_O) [12]. Furthermore, the efficacy of the use of DCV in supraglottic airway devices has not been extensively studied. Ghabach et al., in an observational study, have reported lower PAWP with PCV and PCV-VG than with VCV in patients ventilated using LMA Classic, along with no airway leaks in any ventilation mode in any of the patients [13]. These reports suggest that PCV and DCV are preferable to VCV for regulating PAWP in patients ventilated using supraglottic airway devices [4, 12, 13]. However, the efficacy of PCV in reducing airway leak is not clear. To the best of our knowledge, this is the first report to examine the effects of DCV and VCV on ventilatory parameters and airway leak during laparoscopic procedures that require patients to be in the Trendelenburg position when ventilated using supraglottic airway devices. We found that contrary to our hypothesis that regulated PAWP would decrease airway leak, lower PAWP in the AutoFlow ventilation device did not decrease airway leak compared with VCV.

Patients with an airway leak had lower OLP and a higher number of positive bubble tests than those without an airway leak. As FOS and gastric tube insertion were not related to the incidence of airway leak, we assume that the relationship between the patient’s anatomy and the device was the most likely cause of airway leak rather than malposition of pLMA. It is known that higher PAWP than OLP can cause airway leak during positive pressure ventilation with supraglottic airway devices; therefore, it is recommended to maintain OLP at ≥ 25 cmH_2_O or > 8 cmH_2_O above PAWP during laparoscopic surgery with supraglottic airway devices [7]. This concept is similar to our result that the area under the receiver-operating curve of OLP for its effects on airway leak was high and the sum of its sensitivity and specificity was highest at OLP 23 cmH_2_O. Thus, if patients with OLP < 23 cmH_2_O need to undergo laparoscopic gynecological surgery, careful consideration should be given to whether continued management with pLMA is acceptable or if ventilation should be provided by tracheal intubation. Although endotracheal tubes have been used the most, we believe our results may promote the use of pLMA in patients with OLP > 23 cmH_2_O in laparoscopic gynecological surgery.

The above notwithstanding, there are some limitations to this study. First, we could not blind the attending anesthesiologists to patient randomization. Second, this is a relatively small, single-center study, which was not designed to assess differences in the incidence of airway leak or pulmonary complications, even though it has sufficient power to detect differences in PAWP. A future larger study is needed to compare these outcomes. Third, we did not compare AutoFlow ventilation with PCV, and thus, our study cannot comment on the advantages of DCV over PCV. Additionally, even though DCV can advantageously maintain target tidal volume compared with PCV, future studies should evaluate whether it contributes to reducing pulmonary complications as well. Finally, as we only included healthy adult women with ASA-PS 1 or 2 and normal airways, our findings cannot be extended to patients with a critical illness or morbid obesity.

## Conclusions

In conclusion, while AutoFlow ventilation decreased PAWP compared to VCV during laparoscopic gynecological surgery using pLMA, it could not reduce the incidence of airway leaks.

## Supplementary Information

**Additional file 1.**

## Data Availability

Data are available from the corresponding author on reasonable request.

## Abbreviations

ASA-PS: American Society of Anesthesiologists physical status
DCV: Dual-controlled ventilation
EtCO_2_: End-tidal carbon dioxide
FiO_2_: Fractional inspired oxygen
FOS: Fiberoptic score
OLP: Oropharyngeal leak pressure
PAWP: Peak airway pressure
PCV: Pressure-controlled ventilation
PCV-VG: Pressure-controlled ventilation volume guaranteed
pLMA: LMA ProSeal
VCV: Volume-controlled ventilation

## References

[1] Maltby JR, Beriault MT, Watson NC, Liepert DJ, Fick GH (2003). LMA-classic and LMA-ProSeal are effective alternatives to endotracheal intubation for gynecologic laparoscopy. Can J Anaesth.

[2] Miller DM, Camporota L (2006). Advantages of ProSeal and SLIPA airways over tracheal tubes for gynecological laparoscopies. Can J Anaesth.

[3] Hohlrieder M, Brimacombe J, Eschertzhuber S, Ulmer H, Keller C (2007). A study of airway management using the ProSeal LMA laryngeal mask airway compared with the tracheal tube on postoperative analgesia requirements following gynaecological laparoscopic surgery. Anaesthesia..

[4] Natalini G, Facchetti P, Dicembrini MA, Lanza G, Rosano A, Bernardini A (2001). Pressure controlled versus volume controlled ventilation with laryngeal mask airway. J Clin Anesth.

[5] Assad OM, El Sayed AA, Khalil MA (2016). Comparison of volume-controlled ventilation and pressure-controlled ventilation volume guaranteed during laparoscopic surgery in Trendelenburg position. J Clin Anesth.

[6] Lee JM, Lee SK, Rhim CC, Seo KH, Han M, Kim SY, Park EY (2020). Comparison of volume-controlled, pressure-controlled, and pressure-controlled volume-guaranteed ventilation during robot-assisted laparoscopic gynecologic surgery in the Trendelenburg position. Int J Med Sci.

[7] Timmermann A, Bergner UA, Russo SG (2015). Laryngeal mask airway indications: new frontiers for second-generation supraglottic airways. Curr Opin Anaesthesiol.

[8] Keller C, Pühringer F, Brimacombe JR (1998). Influence of cuff volume on oropharyngeal leak pressure and fibreoptic position with the laryngeal mask airway. Br J Anaesth.

[9] Brimacombe J, Berry A (1993). A proposed fiber-optic scoring system to standardize the assessment of laryngeal mask airway position. Anesth Analg.

[10] Keller C, Brimacombe JR, Keller K, Morris R (1999). Comparison of four methods for assessing airway sealing pressure with the laryngeal mask airway in adult patients. Br J Anaesth.

[11] Bagchi A, Rudolph MI, Ng PY, Timm FP, Long DR, Shaefi S, Ladha K, Vidal Melo MF, Eikermann M (2017). The association of postoperative pulmonary complications in 109,360 patients with pressure-controlled or volume-controlled ventilation. Anaesthesia..

[12] Jeon WJ, Cho SY, Bang MR, Ko SY (2011). Comparison of volume-controlled and pressure-controlled ventilation using a laryngeal mask airway during gynecological laparoscopy. Korean J Anesthesiol.

[13] Ghabach MB, El Hajj EM, El Dib RD, Rkaiby JM, Matta MS, Helou MR (2017). Ventilation of nonparalyzed patients under anesthesia with laryngeal mask airway, comparison of three modes of ventilation: volume controlled ventilation, pressure controlled ventilation, and pressure controlled ventilation-volume guarantee. Anesth Essays Res.

