# Supplementary material for: AutoFlow® versus volume-controlled ventilation for laparoscopic gynecological surgery using LMA® ProSeal™: a randomized controlled trial
Source: BMC Anesthesiol. 2021 Jun 28;21:181. doi: 10.1186/s12871-021-01406-6 (PMC8237450; doi:10.1186/s12871-021-01406-6)
Supplement: Supplementary file 1 — Additional file 1. [file 12871_2021_1406_MOESM1_ESM.docx]

**Supplementary Table**

The other ventilatory parameters and vital signs

|  | AutoFlow group (n=40) | VCV group (n=39) | P value |
| --- | --- | --- | --- |
| 1. FiO_2_ | 0.34 [0.32–0.41] | 0.35 [0.32–0.40] | 0.87 |
| 2. FiO_2_ | 0.31 [0.30–0.39] | 0.33 [0.31–0.40] | 0.069 |
| 3. FiO_2_ | 0.31 [0.30–0.39] | 0.32 [0.30–0.40] | 0.22 |
| 4. FiO_2_ | 0.30 [0.29–0.39] | 0.31 [0.30–0.40] | 0.15 |
| 1. EtCO_2_ (mmHg) | 38 [37–40] | 39 [36–42] | 0.73 |
| 2. EtCO_2_ (mmHg) | 37 [36–39] | 37 [36–40] | 0.71 |
| 3. EtCO_2_ (mmHg) | 36 [35–38] | 35 [34–38] | 0.44 |
| 4. EtCO_2_ (mmHg) | 37 [36–39] | 36 [35–40] | 0.25 |
| 1. EtSev (%) | 1.4 [1.3–1.5] | 1.3 [1.2–1.5] | 0.46 |
| 2. EtSev (%) | 1.3 [1.3–1.4] | 1.3 [1.2–1.4] | 0.186 |
| 3. EtSev (%) | 1.3 [1.3–1.4] | 1.3 [1.2–1.4] | 0.60 |
| 4. EtSev (%) | 1.3 [1.2–1.3] | 1.3 [1.2–1.3] | 0.53 |
| 1. Respiratory rate (/min) | 12 [12–13] | 12 [11–13] | 0.37 |
|  | AutoFlow group (n=40) | VCV group (n=39) | P value |
| 2. Respiratory rate (/min) | 12 [12–13] | 12 [12–14] | 0.97 |
| 3. Respiratory rate (/min) | 12 [12–14] | 13 [12–14] | 0.66 |
| 4. Respiratory rate (/min) | 14 [12–14] | 13 [12–14] | 0.53 |
| 1. leak fraction (%) | −2.3 [−4.2 to −0.1] | −2.8 [−5.5 to −0.7] | 0.28 |
| 2. leak fraction (%) | −1.5 [−3.7 to 0.5] | −2.5 [−5.1 to −0.1] | 0.26 |
| 3. leak fraction (%) | −2.2 [−3.8 to −0.2] | −2.6 [−4.8 to −0.3] | 0.92 |
| 4. leak fraction (%) | −1.6 [−5.2 to 0.8] | −1.9 [−4.3 to −0.6] | 0.47 |
| 1. SpO_2_ (%) | 99 [98–100] | 99 [98–100] | 0.70 |
| 2. SpO_2_ (%) | 99 [98–100] | 99 [98–100] | 0.81 |
| 3. SpO_2_ (%) | 99 [98–100] | 99 [98–100] | 0.98 |
| 4. SpO_2_ (%) | 99 [98–100] | 99 [98–100] | 0.91 |
| 1. Heart rate (/min) | 60 [51–63] | 58 [53–64] | 0.78 |
| 2. Heart rate (/min) | 59 [53–63] | 57 [53–65] | 0.87 |
| 3. Heart rate (/min) | 59 [55–64] | 59 [56–69] | 0.59 |
| 4. Heart rate (/min) | 60 [57–64] | 61 [54–70] | 0.86 |
| 1. SBP (mmHg) | 83 [78–90] | 84 [80–92] | 0.57 |
|  | AutoFlow group (n=40) | VCV group (n=39) | P value |
| 2. SBP (mmHg) | 85 [79–92] | 82 [77–90] | 0.49 |
| 3. SBP (mmHg) | 85 [81–91] | 84 [78–90] | 0.28 |
| 4. SBP (mmHg) | 91 [87–100] | 89 [83–97] | 0.31 |
| 1. DBP (mmHg) | 52 [46–56] | 54 [48–58] | 0.30 |
| 2. DBP (mmHg) | 52 [48–58] | 52 [47–59] | 0.63 |
| 3. DBP (mmHg) | 56 [50–63] | 56 [48–61] | 0.62 |
| 4. DBP (mmHg) | 59 [54–68] | 60 [54–65] | 0.94 |
| 1. MBP (mmHg) | 62 [57–67] | 64 [56–69] | 0.63 |
| 2. MBP (mmHg) | 63 [60–67] | 62 [57–69] | 0.33 |
| 3. MBP (mmHg) | 66 [60–72] | 64 [57–70] | 0.187 |
| 4. MBP (mmHg) | 69 [63–77] | 69 [63–79] | 0.90 |

1, One min after insertion of the gastric tube; 2, Two min after intravenous administration of neuromuscular blocking drug; 3, One min after initiation of pneumoperitoneum; 4, One min after change to the Trendelenburg position. Data are shown as median [interquartile range].

FiO_2_: Fractional inspired oxygen, EtCO_2_: end-tidal carbon dioxide, EtSev: end-tidal sevoflurane, SBP: systolic blood pressure, DBP: diastolic blood pressure, MBP: mean blood pressure
